# Update of application of olfactory ensheathing cells and stem cells/exosomes in the treatment of retinal disorders

**DOI:** 10.1186/s13287-021-02685-z

**Published:** 2022-01-10

**Authors:** Yang Yu, Licheng Li, Shu Lin, Jianmin Hu

**Affiliations:** 1 Department of Ophthalmology, The Second Affiliated Hospital of Fujian Medical University, Engineering Research Center of Assistive Technology for Visual Impairment, Fujian Province University, Quanzhou, 362000 Fujian Province China; 2 Centre of Neurological and Metabolic Research, The Second Affiliated Hospital of Fujian Medical University, Quanzhou, 362000 Fujian Province China; 3 Diabetes and Metabolism Division, Garvan Institute of Medical Research, 384 Victoria Street, Darlinghurst, Sydney, NSW 2010 Australia; 4 The School of Medical Technology and Engineering, Fujian Medical University, Fuzhou, 350004 Fujian Province China

**Keywords:** Retinal disorders, Olfactory ensheathing cells, Stem cells, Exosomes

## Abstract

Age-related macular degeneration, diabetic retinopathy, retinitis pigmentosa and other retinal disorders are the main causes of visual impairment worldwide. In the past, these retinal diseases, especially dry age-related macular degeneration, proliferative diabetic retinopathy and retinitis pigmentosa, were treated with traditional surgery and drugs. However, the effect was moderate. In recent years, researchers have used embryonic stem cells, induced pluripotent stem cells, mesenchymal stem cells, olfactory ensheathing cells and other stem cells to conduct experiments and found that stem cells can inhibit inflammation, regulate immune response, secrete neurotrophic factors, and differentiate into retinal cells to replace and promote restoration of the damaged parts. These stem cells have the potential to treat retinal diseases. Whether it is in animal experiments or clinical trials, the increase in the number of retinal cells, maintenance of function and improvement of visual function all reflect the advanced of stem cells to treat retinal diseases, but its risk preserves the donor’s hidden pathogenic genes, immune rejection and tumorigenicity. With the development of exosomes study, researchers have discovered that exosomes come from a wide range of sources and can be secreted by almost all types of cells. Using exosomes with stem cell to treat retinal diseases is more effective than using stem cells alone. This review article summarizes the recent advances in the application of olfactory ensheathing cells and stem cells/exosomes in the treatment of retinal disorders.

## Introduction

Cardiovascular diseases, tumors and visual impairment are the three major diseases that affect the quality of life of people all over the world. According to the survey results of the World Health Organization (WHO), by 2020, there will be approximately 553 million visually impaired people in the world. Among them, about 295 million people suffer from moderate and severe visual impairment, and about 43 million people are blind. By 2050, this number will increase to 61 million people who are blind and 474 million people have moderate to severe visual impairment. In 2020, the main cause of visual impairment for people aged 50 and over in the world is cataract, followed by glaucoma, uncorrected refractive error, age-related macular degeneration (AMD), and diabetic retinopathy (DR) [1]. Among these main causes, glaucoma, AMD, and DR, like retinitis pigmentosa (RP) and Stargardt's disease, all present different types of retinopathy, causing patients with different degrees of visual impairment, and bringing about great changes in patients` study, work and life.

Visual impairment not only brings great pain to patients and their families, but also causes different degrees of social and economic burden. For the retinopathy that seriously affects the quality of human life, researchers from all over the world are actively popularizing the knowledge of related diseases and researching on diagnosis and treatment methods. At present, besides surgery, drugs and gene therapy, stem cell therapy is also a novel research direction. In this review, we will explore the potential of olfactory ensheathing cells and stem cells/exosomes applications in retinal disorders.

## Retinal disorders

### Age-related macular degeneration (AMD)

#### AMD and its treatment

AMD is a chronic progressive retinal disease characterized by a progressive and irreversible decline in central vision. It mainly affects macula, retinal pigment epithelial (RPE) and choroid. The clinic is mainly divided into two categories: dry AMD (also known as atrophic AMD) and wet AMD (also known as exudative AMD). Dry AMD is usually characterized by slow and progressive decline in vision, map atrophy of the RPE of the macula, atrophy of choroidal capillaries, and the formation of drusen. Wet AMD can rapidly change vision and lose central vision within a few weeks to several months, mainly characterized by the formation of choroidal neovascularization (CNV), and a series of pathological changes such as secondary exudation, hemorrhage and scarring [2, 3]. The pathogenesis of AMD is unclear, and may involve RPE damage, mitochondrial dysfunction, oxidative stress, inflammation, or activation of the complement pathway [4–6].

Currently, there is no specific treatment for AMD, especially for dry AMD. Commonly used treatment methods include laser photocoagulation, surgery and drug therapy. In the clinical treatment of AMD, a variety of therapeutic methods are generally used in combination. Laser photocoagulation can only be used for AMD where the location of the neurovascular injury is well defined and not in the fovea, and may result in impaired vision. Currently, anti-vascular endothelial growth factor (anti-VEGF) is mainly used for the treatment of wet AMD [7, 8]. However, long-term use of anti-VEGF will not only reduce the efficacy, but may also lead to retinal hemorrhage, fibrosis and scarring, macular atrophy or retina map atrophy [9, 10]. Researchers have reported the use of antioxidant enzymes to treat AMD. Studies have shown that these enzymes can alleviate the adverse effects of oxidative stress on RPE and photoreceptors [11, 12]. If patients with AMD have vitreous hemorrhage, vitreoretinal surgery requires rapid removal of blood and subretinal neovascular membrane.

### Animal model of AMD

#### Animal model of wet AMD

By injecting substances capable of expressing VEGF under the retina, a wet AMD animal model can be established [13, 14]. Sherpa et al. reported that the non-human primate models of AMD could be established by using adeno-associated virus (AAV) VEGF to promote neovascularization in rhesus monkeys [15]. Injection of RPE or RPE and polystyrene microbeads or polyethylene glycol under the retina of C57BL/6 mice and delt ACCL-2 mice can induce CNV formation [16, 17]. Of course, subretinal injection of macrophages and lipid peroxides can also establish animal models of wet AMD [18, 19]. Laser can selectively destroy the outer segment of photoreceptors, RPE, Bruch’s membrane and choroidal capillaries, causing a series of damage and repair reactions, and then forming new blood vessels [20]. Olvera-montano et al. reported that the non-human primate model of AMD was successfully established if more than one point with fluorescence leakage of grade III or IV lesions occurred after laser irradiation near macular area of macaque monkeys [21]. Alternatively, wet AMD can also be modeled by animals with specific genotypes. Studies have shown that CCL-2 and CCR-2 knockout mice have pathological changes similar to human AMD [22]. The chemokine receptor 1 antibody knockout (CX3CRL−/−) mouse model can be used to study the correlation between retinal microglia and the pathological mechanism of AMD [23]. A series of early pathological manifestations of AMD were observed in transgenic human Apob100 mice after 12 months [24]. In the study of Sun et al., circRims2 was knocked down with short hairpin RNA through subretinal AAV in mice, and the thickness of the outer and inner segment layers and outer nuclear layer (ONL) were reduced [25]. Chen et al. found that silencing circTulp4 resulted in thin ONL and retinal dysfunction in mice [26]. Researcher reported that the expression of lncRNA ZNF503-AS1 was down-regulated in the RPE-choroid of AMD patients [27]. Chen et al. demonstrated that both silencing and overexpression of Ago2 in mouse retina led to thinning of the ONL and shortening of the outer and inner segments, which was also reflected in the weakened electroretinogram (ERG) response. In addition, the destruction of Ago2 also resulted in changing the non-coding RNAs in the retina [28]. If these findings can be used to edit corresponding miRNA, lncRNA or circRNA in animals, some new methods for establishing AMD animal models may be obtained.

#### Animal model of dry AMD

Sod1 (−/−)mice produced oxidative damage to tissues. Thus, it can be seen that this mouse could be used as an ideal model for the study of dry AMD, especially for the study of oxidative stress response and the pathogenesis of AMD [29]. Another animal model of dry AMD, senescence accelerated mouse (SAM), showed functional senescence in various parts of the body early in life, which should only occur in old age. AMD-like changes were observed in the 10-month-old SAMP8 mice [30].

There is no macular area in the retina of rodents. The mechanism of various pathological changes may be different from the natural onset of human AMD. However, some mouse and rat models have inherent genetic defects, leading to the absence of certain signal transduction and metabolic pathways. For these reasons, caution should be taken when selecting such animals for AMD research.

### Diabetic retinopathy (DR)

#### DR and its treatment

DR refers to ocular retinopathy induced by metabolic disorders caused by elevated blood sugar in diabetic patients. According to the degree of damage to the fundus, DR is classified as nonproliferative diabetic retinopathy (NPDR) and proliferative diabetic retinopathy (PDR). NPDR is characterized by vascular tortuosity, retinal hemorrhage, micro hemangioma, and lipid exudation. When abnormal new blood vessels appear, NPDR develops into PDR. Another important feature of DR is diabetic macular edema (DME), which is caused by retinal thickening and cystic macular changes caused by fluid accumulation in the nerve retina. DME can occur in NPDR or PDR and is a common cause of vision loss. The pathological changes of DR are mainly retinal capillary endothelial damage, including loss of selective pericytes, thickening of basement membrane, capillary occlusion and leakage due to impaired blood-retinal barrier function. These lead to extensive retinal ischemia, retinal edema and neovascularization in the late stage. The occurrence and development of DR are closely related to the course of diabetes. Studies have shown that the course of diabetes is a risk factor for DR, and the prevalence of DR increases with the course of the disease [31].

For the treatment of DR, the first step is to control the systemic situation. Drug treatment, calcium hydroxybenzene sulfonate, can reduce the leakage of retinal blood vessels and improve the fundus microcirculation. Triamcinolone acetonide is a long-acting corticosteroid that can be used for the treatment of DME. Anti-VEGF plays an important role in the occurrence and development of DME and DR neovascularization [32]. Retinal photocoagulation uses laser thermal coagulation to destroy RPE cells, reduce retinal oxygen consumption, prevent the occurrence of new blood vessels, and promote new blood vessel atrophy. Vitrectomy is the most effective method for the treatment of vitreous hemorrhage and retinal detachment during DR proliferative stage.

#### Animal model of DR

At present, DR animal models are based on diabetic animal models. Clinically, hyperglycemia is caused by the injection of streptozotocin (STZ) or alloxan. The number of astrocytes increased in diabetic mice after 4 to 5 weeks of elevated blood glucose [33]. The diabetic mice began to develop the early characteristic of DR 6 months after the onset of the hyperglycemia [34]. Non obese diabetes (NOD) mice may be a useful animal model for studying juvenile diabetes in humans. At 30 weeks of age, about 80% of female NOD mice and 20% of male NOD mice developed hyperglycemia [35]. Studies had shown that Lewis rats treated with STZ exhibited retinal capillary and retinal ganglion cells (RGCs) loss after 8 months of elevated blood glucose [36]. Similarly, in addition to the development of DR by raising blood glucose levels in rabbits with STZ or alloxan injections, retinal microaneurysms in New Zealand rabbits could be induced by feeding a diet high in fat, sucrose and cholesterol [37]. STZ could induce diabetes in monkeys, and changes in cotton-wool spots and high-fluorescence spots could be observed on the retina [38]. After hypoxia intervention, the monkeys showed vascular leakage, venous occlusion, capillary nonperfusion, venous dilatation, and dot and blot hemorrhages. This method could be used to establish a non-human primate animal model of DR [38]. Rhesus monkeys were intravitreal injected with an AAV-2 vector carrying human VEGF gene to observe the formation of retinal neovascularization [39]. MALAT1, an important lncRNA, was an epigenetic regulator of many inflammatory cytokines in DR. It reduced microvascular injury and inflammation associated with DR. It has been proved that knocking down MALAT1 could reduce reactive gliosis and Müller cell activation [27]. The lncRNA NEAT1 expressed in RPE had been shown to delay the development of DR in diabetic mouse models [40]. Jiang et al. reported that circRNA cZNF532 was up-regulated in retinal vessels of diabetic mice and in the vitreous fluid of diabetic patients. Then they knocked out cZNF532 in a STZ induced animal model of diabetes and found that the retinal pericyte degeneration and vascular dysfunction worsened [41].

Up to now, there is no single model that can imitate the whole process of human DR development. Rodents have been widely used in DR studies because of their small size and their ability to study the manifestations of retinopathy over a relatively short period of time. However, most rodent DR models only show early symptoms of DR. Some advanced animals show deeper retinopathy, but they still cannot mimic the late-stage symptoms of human DR. In addition, high maintenance costs and long research time limit its further application.

### Retinitis pigmentosa (RP)

#### RP and its treatment

RP is a genetic disorder that can cause low vision and even blindness. It is characterized by the progressive loss of function of photoreceptors and RPE. The inheritance modes of RP mainly include autosomal dominant inheritance, autosomal recessive inheritance and X-linked inheritance. A small number of RP is inherited by mitochondria and dihybrid inheritance, and there are also sporadic cases. At present, about 80 mutant genes related to non-syndromic RP have been discovered. Another 12 mutated genes are related to Usher syndrome, and 17 mutated genes are related to Bardet-Biedl syndrome [42]. The initial symptoms of RP include nocturnal vision loss and dark adaptation disorder, but it may also manifest as early mid-peripheral visual field defects, followed by concentric visual field loss. The typical fundus abnormalities are bone spicule pigmentation, narrowed retinal vessels, and waxy paleness of the optic disc. In addition, some patients with RP will have macular cystic edema [43], posterior cystic cataract [44], and anterior retinal membrane [45].

The treatment of RP can be done through gene therapy, cell transplantation, retinal prosthesis and drug therapy. Bcl-2 gene were introduced into the retina of rd6 mice, and it was found that it can effectively maintain the structure and function of the retina [46]. RP can be treated by transplantation of retinal progenitor cells, embryonic stem cells and induced pluripotent stem cells. It has been reported that patients with light perception can use the visual prostheses, Argus II system, to recognize letters and words [47]. As for the possibility of using medicine to treat RP, vitamin A palmitate could slow down the loss of cones function in children with RP [48]. Docosahexaenoic acid, taurine and traditional Chinese medicine have been reported in the treatment of RP, but the efficacy is still unclear.

#### Animal model of RP (Table 1)

**Table 1:** Common animal models of RP

Classification	Common animal models	Characteristics	References
Natural	rd1 mouse	Autosomal recessive	Kalloniatis et al. [49]
	RCS rat	Autosomal recessive	He et al. [50]
	Cat	Autosomal recessive	Winkler [51]
	Dog	Autosomal recessive, RPE65 genetic mutations	Dinculescu et al. [52]
	Mouse and dog	Autosomal dominant, RHO genetic mutations	Massengill et al. [53]
	Mouse	X-linked, RPGR deficiency	Gumerson et al. [54]
Drug injection	Rat	N-ethyl-N-nitrosourea Intraperitoneal injection	Yoshizawa et al. [56]
	Sheep	Sodium iodate intravenous injection	Ong et al. [55]
Laser	Rat	Blue light exposure	Vila et al. [57]
Gene knock-out	Mouse	RP2 knock-out	Mookherjee et al. [58]
	Mouse	CNGB1 knock-out	Michalakis et al. [59]
	Mouse	SPATA7 knock-out	Zhong et al. [60]
	Rat	LRAT knock-out	Koster et al. [61]
	Mouse	miRNA-183/96 knock-out	Xiang et al. [62]
	Mouse	miRNA-183 knock-out	Zhang et al. [63]
	Mouse	miRNA-182 knock-out	Wu et al. [64]
Gene knock-in	Mouse	P23H opsin knock-in	Sakami et al. [65]
	Mouse	RhoD190N knock-in	Sancho-Pelluz et al. [66]

RP had a natural animal model. Rd1 mouse was an autosomal recessive RP animal model [49]. RCS rats had recessive mutations in the Mertk gene of receptor tyrosine kinase [50]. Early-onset autosomal recessive RP was found in cats [51]. Dogs had congenital RP, RPE65 genetic mutations [52]. Rhodopsin (RHO) gene mutations had been widely used in mice and dogs as animal models of autosomal dominant RP [53]. Gumerson et al. established an X-linked RP animal model, which was a mouse model of retinitis pigmentosa GTPase regulator (RPGR) deficiency similar to human RP3 [54]. Researchers used drug injection to create animal models of RP. Intravenous injection of sodium iodate had a significant destructive effect on the structure of sheep RPE. Progressive, photoreceptors, Muller cells and RGCs may be damaged in different degrees [55]. In addition, Yoshizawa et al. reported that N-ethyl-N-nitrosourea could induce mutations in the Pde6b gene, which was another drug injection method for establishing RP animal models [56]. Laser at 380 nm would damage rat photoreceptors, while laser at 470 nm caused damage to both the photoreceptors and RPE. Therefore, blue light can be used to irradiate rats to establish a RP model [57]. Gene-editing techniques can also be used to create models. Some of the RP animal studies were conducted using gene knock-out animals such as RP2 knock-out mouse, CNGB1 knock-out mouse, SPATA7 knock-out mouse and LRAT knock-out rat [58–61]. Previous studies had proved that miRNA gene mutations caused hereditary retina degeneration such as RP. The loss of miRNA-183/96 led to changes in the retina, such as thinning of ONL, activation of microglia and narrowing of blood vessels, which eventually resulted in a gradual decrease or even disappearance of the ERG response [62]. Similarly, Zhang et al. reported that knocked out miRNA-183 in mice, and the ERG examination results of these mice showed that the response amplitude was gradually weakened [63]. When miRNA-182 was knocked out, the ERG response amplitude of these non-coding gene knockout mice was decreased at an early stage, and the expression of some key photoreceptor specific genes was down-regulated with the loss of miRNA-182 in the retina [64]. In addition, knocking in the P23H opsin and RhoD190N genes in mice could also observe the pathological mechanism of RP [65, 66].

**Fig. 1:**
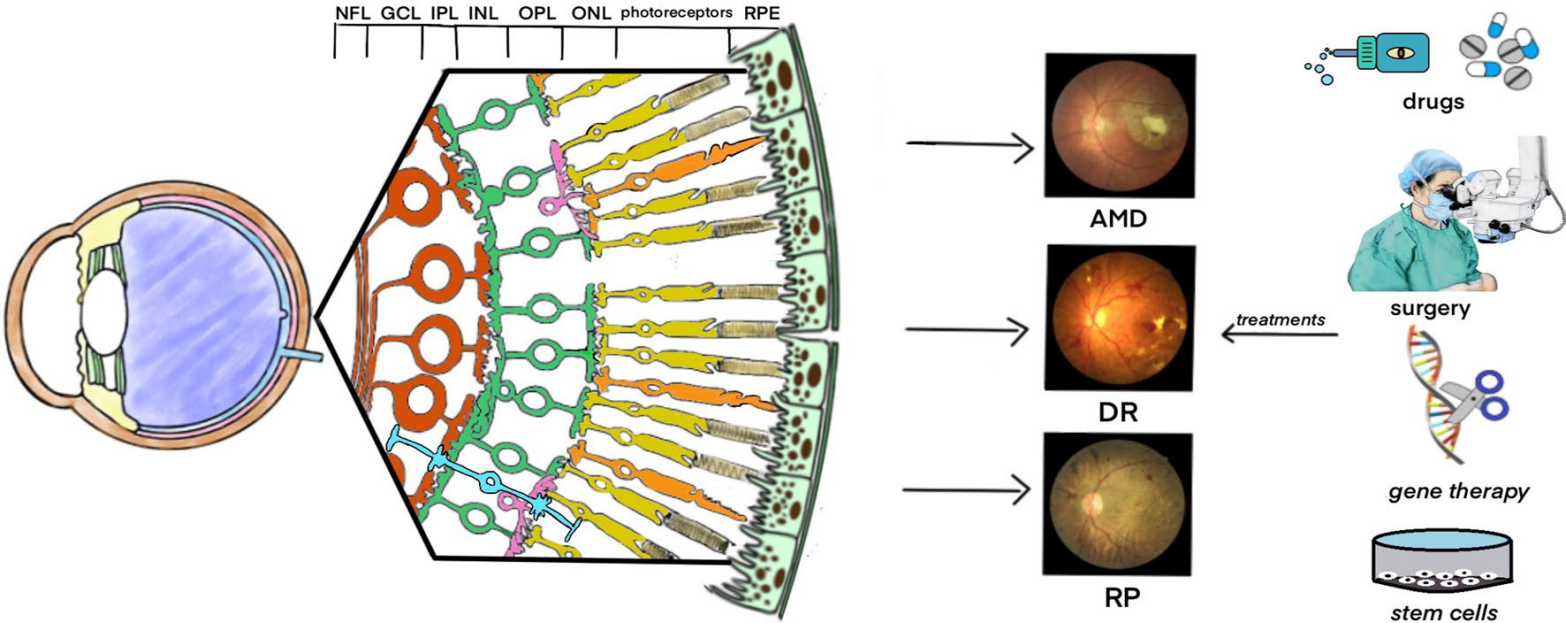
Retinal disorders and treatments. The normal retina is made up of a variety of cells. Common retinal disorders such as AMD, DR, RP, fundus photography will show a map atrophy, retinal hemorrhage and exudation, bone spicule pigmentation. Currently, drugs, surgery, gene therapy and stem cells are used to treat these retinal disorders

Figure 1 summarizes common retinal disorders and treatments.

### Stem cells include olfactory ensheathing cells (OECs) used to treat retinal diseases

In recent years, with the rapid development of science and technology, the treatment of stem cells including OECs has gradually become a clinical treatment method with broad application prospects and good therapeutic effects. In addition to the use of stem cells to treat spinal cord injury, Parkinson's disease, myocardial infarction and wound repair, stem cells are also used in ophthalmic diseases, such as optic nerve injury, AMD, DR, RP, Stargardt's disease is also being gradually developed in preclinical and clinical studies.

### OECs

The olfactory neurons of adult mammals are renewed regularly throughout their lives [67]. Part of the reason for the regenerative expression of this special olfactory system is believed to be the existence of a special type of glial cell, OECs [68].

### Distribution of OECs

OECs originate from the olfactory lamina, migrate from lamina propria to the olfactory epithelium along the axons, and cross the cribriform plate into the cranial cavity, finally forming glial cells surrounding the olfactory bulb [69]. OECs are generally distributed in the olfactory nerve fiber layer and the glomerular layer of olfactory bulb and the olfactory mucosa [70]. In humans, although OECs can be obtained from both olfactory mucosa and olfactory bulb, the olfactory bulb is located in the skull, so obtaining OECs from the olfactory bulb requires a high risk of craniotomy. The olfactory mucosal tissue mass does not require craniotomy, and only needs to be clipped by nasal endoscope under local anesthesia. Therefore, it is easier and safer to obtain autologous human OECs from the olfactory zone of the nasal mucosa, and the OECs from the upper turbinate are more than those from the inner side of the middle turbinate.

### Morphology and classification of OECs

Some studies have shown that there are different types of OECs subgroups in the olfactory system [69]. The materials expressed by OECs from the olfactory bulb and lamina propria are significantly different. The OECs in the olfactory bulb express p75, S100β, GFAP, and O4. However, OECs in the lamina propria express a unique combination of important developmental proteins, including CD44, β1 integrin, P200, Notch3, NG2, VEGF, PACAP and CREB binding proteins, which are not expressed in the olfactory bulb [71].

### The function of OECs

OECs can promote neuron survival and axon growth, coordinate and directly extend axons to the target area. Injection of OECs into the cross-section of the rat spinal cord can promote axon regeneration, which is shown to improve hind limb function in rats [72]. After sciatic nerve transection transplantation in rats, it was found that OECs can promote the regeneration of injured nerves and improve their functions [73]. After middle cerebral artery occlusion rats were transplanted with OECs around the basal ganglia infarction, they were found to have a protective effect on the ischemic damage of white matter [74].

OECs can secrete p75NGF receptor, nerve growth factor (NGF), glial cell lineage-derived neurotrophic factor (GDNF), brain-derived neurotrophic factor (BDNF), neuropeptide Y (NPY), S100 and other neurotrophic factors and extracellular matrix components. This characteristic of OECs can improve the microenvironment for the growth of damaged neurons and axons. This not only can effectively promote neuronal survival and axon growth in spinal cord injury, but also may help axons extend long distances to damaged areas of the central nervous system [75–79]. OECs can also express a variety of immune factors, such as alpha-melanocyte stimulating hormone [80], and have the function of continuously phagocytosis of bacteria and apoptotic nerve cell fragments [81, 82]. OECs take advantage of these characteristics to create a favorable environment for the regeneration and repair of neurons and axons by regulating the immune response and phagocytosis.

Overall, OECs can improve the cell growth microenvironment through their own proliferation and migration, or by secreting different types of nutritional factors and immune factors, so as to achieve the repair effect of damaged nerves.

### Treatment of OECs

Yin et al. demonstrated that OECs could protect damaged neurons at the site of optic nerve injury by injecting concentrated OECs suspension [83]. Wu et al. found that transplantation of OECs into the eye stump of rats where the optic nerve was completely severed resulted in higher survival rate of the RGCs than the group without OECs, and the expression of BDNF in the eye stump and retina could also be induced [84]. Research by Huo et al. showed that transplanted OECs could inhibit the glial injury response of Muller cells in a rat model of RP [85]. This team also reported that after transplantation of OECs and olfactory nerve fibroblasts into the subretinal cavity of rats with RP, they could migrate to other retinal layers and removed the accumulated photoreceptors outer segment debris in the subretinal cavity, and expressed and secreted NGF, BDNF and basic fibroblast growth factor (BFGF), which contributed to the survival of photoreceptors [86]. Similar results were found by Xie et al. that the transplantation of OECs in the experimental animals could improve the retinal immune environment, inhibit the activation of Muller cells, and protect photoreceptor cells and bipolar cells [87]. The researchers transplanted OECs into a laser induced retina degeneration rat model. These OECs inhibited the retinal oxidative stress response, thereby preserving the structure and function of the retina [88]. Xie et al. also reported similar effects [89]. OECs down-regulates the JAK2/STAT3 pathway. The cell transplantation area activated microglia and the expression of pro-inflammatory cytokines was significantly reduced, while the expression of anti-inflammatory cytokines was significantly increased. Finally, OECs protect the neurons and photoreceptors in the retina. In one study, both OECs and α-crystalline inhibited the apoptosis of RGC-5 cells damaged by hydrogen peroxide, and decreased the activation of caspase-3. Furthermore, the combined use of OECs and α-crystallin was more meaningful than its used alone [90]. In exploring the effect of combined treatment with OECs, Zhai et al. revealed that the combined OECs and neural stem cells (NSCs) transplantation group enhanced cell migration ability, significantly increased the thickness of the retinal outer nuclear layer, and increased the number of endogenous stem cells in the retina [91].

Summary up, OECs also play an important role in the treatment of optic nerve injury and retinal degeneration, which are similar to spinal cord injuries and degenerative brain diseases. Transplanting OECs on the stump of optic nerve or retina can not only increase the activity of the residual RGCs and promote their regeneration, but also improve the cell living environment and protect the photoreceptors.

### Embryonic stem cells (ESCs)

ESCs are derived from a large number of cells in the inner layer of the blastocyst embryo. They have the ability to self-renew and differentiate into any type of cell in the body. In some experiments to study the treatment of retinal degenerative diseases, researchers have developed the capabilities of ESCs. By making certain inducing conditions, the researchers induced the ESCs to differentiate into RGCs, RPE and photoreceptors, and then transplanted the differentiated cells into the animal models to replace damaged cells. Leach et al. successfully induced ESCs to express RPE markers. These ESCs could phagocyte extracellular segments of photoreceptors, and secrete cytokines pigment epithelial-derived factors and VEGF, which were consistent with the functions of RPE in vivo [92]. Another study demonstrated that the RPE patch derived from ESCs could be used to treat AMD [93]. Retinal neurons derived from human ESCs which were injected into the submacular of squirrel monkeys had survived for at least three months, and the researchers observed that some of the donor cells integrated into the inner retina and that many of the neurons' axons projected into the optic nerve [94]. Another study transplanted human ESCs-derived RPE into submacular of a macaques. After transplantation, human ESCs derived RPE exhibited phagocytosis in vivo, and photoreceptors near the transplanted area were preserved, which also showed the preservation of amplitude and peak time in ERG [95]. However, there are still some technical difficulties in directed differentiation and culture. These ESCs have the risk of inducing tumors and will face immune rejection. As for the genetic defect that may exist in the allogeneic donor, the defect is also an unavoidable problem when using the ESCs therapy.

It can be seen that researchers mainly use the differentiation ability of ESCs to induce specific cell types such as RPE, and use the differentiated cells to replace the original damaged cells. However, attention should be paid to the risk of tumorigenicity and possible immune response and genetic defects.

### Mesenchymal stem cells (MSCs)

MSCs are a kind of multifunctional stromal cells. These cells are mainly derived from bone marrow, but can also be obtained from adipose tissue, placenta, umbilical cord blood, dental pulp, heart muscle, and liver. MSCs have the function of self-renewal and differentiation. They mainly differentiate into mesoderm derived tissues, but also into endoderm and ectoderm derived tissues. MSCs have the function of paracrine nutrition and can secrete neurotrophic factors and angiogenic factors, such as ciliary growth factor, BFGF, VEGF and so on. Scalinci et al. found that in diabetic animal models, after intravitreal injection of MSCs, the density of neuroprotective factors in the retina increased, which can significantly inhibit RGCs apoptosis [96]. The ability of MSCs to differentiate into damaged tissues is also limited. In diabetic animal models, local or systemic injection of MSCs can reduce the degree of disease through differentiation into photoreceptors and RPE [97, 98]. In addition, MSCs also have immunomodulatory functions. It can inhibit the differentiation of monocytes in vivo to macrophages, increase the level of IL-10, decrease the levels of IL-12 and IFN-γ, inhibit the proliferation of killer T cells, and increase the number of regulatory T cells [99–101]. Chen et al. reported that inflammatory response and caspase-3 protein expression were reduced after adipose-derived MSCs transplantation [102]. The results of Ortiz-Virumbrales M et al. revealed that adipose-derived MSCs could regulate the differentiation of myeloid cells by PGE2 and IL-6 mediating, produce anti-inflammatory and repair effects [103]. Peng et al. found that the MSCs could be induced to differentiate into RPE, which also had the function of phagocytosis of photoreceptor membrane disks [104].

At present, the application of MSCs in the treatment of retinal diseases is a hot spot. MSCs are widely available and can differentiate into damaged retinal tissue cells, as well as nutritional protection of retinal tissue and mitigation of the immune response.

### Induced pluripotent stem cells (iPSCs)

IPSCs reprogram somatic cells into a pluripotent state by directly introducing specific genes, specific gene products or small molecule compounds [105]. Such cells can be extracted directly from the patient's own tissues, and they face fewer ethical issues than other stem cells from external sources. Similar to ESCs, iPSCs can effectively differentiate into various types of retinal cells, including photoreceptors, RGCs, and RPE, by combining soluble factors in a specific environment and an at appropriate time [106–108]. Dohoon and others had shown that iPSCs culture and differentiation into autologous RPE and transplantation can effectively improve the vision of patients with diabetic macular edema [109]. AAV vectors had demonstrated their role as gene therapy vectors for a wide range of retinal disorders. A research showed that ShH10 and AAV2/5 were effective vectors for the differentiation of iPSCs into RPE, while AAV2/8 and ShH10 achieved the differentiation of human ESCs-derived photoreceptors [110]. Michele et al. used two-dimensional culture combined with a classification method based on lipoprotein absorption to study pure RPE. This method could obtain purely functional RPE from iPSCs in a short time, which improved the clinical efficacy of treating retinal disorders [111]. Lingam et al. injected iPSCs-derived photoreceptor precursors into the non-human primate model of retinal degeneration induced by cobalt chloride. Optical coherence tomography was used to demonstrate the restoration of retinal ellipsoid region after transplantation. The graft was still alive three months after transplantation and had the ability to mature into cone photoreceptors [112]. Due to their own source, iPSCs have a low risk of rejection, but because they retain the epigenetic characteristics of the original tissue, they are very likely to carry defective genes from the body.

From previous studies on iPSCs, it can be seen that these cells are extracted from the patient’s own tissues, which has a low risk of rejection and has obvious advantages. However, precisely because iPSCs are extracted from the body, the possibility of carrying disease genes has become a concern.

### Retinal progenitor cells (RPCs)

Retinal progenitor cells (RPCs) are neural progenitor cells (NPCs) located in the inner layer of the optic cup. Rat retinal cells in different gestational stages can differentiate into a variety of retinal cells [113]. Human retinal progenitor cells (hRPCs) are derived from the retina of a 16–20-week-old fetus. HRPCs have the ability to self-renew and differentiate into all retinal cell types under specific conditions [114]. Intravitreal injection of hRPCs can effectively and safely protect the photoreceptors of RCS rats and release a variety of neurotrophic factors [115]. Liu et al. found that subretinal injection of hRPCs in RP patients can repair their damaged retinal cells and promote the secretion of neuroprotective factors. The long-term safety of RPCs transplantation therapy has been confirmed [116].

It can be concluded from previous studies that RPCs have a relatively low risk of immune rejection and tumorigenicity. However, RPCs have a limited ability to proliferate and differentiate into specific target cells. The main problem with RPCs for clinical use is the lack of sufficient donor cells [117]. By improving the proliferation ability of RPCs, the problem of insufficient donor cells can be solved, so as to achieve the effect of wide clinical application.

### Human amniotic epithelial stem cells (hAESCs)

Human amniotic epithelial stem cells (hAESCs) develop from the pluripotent inner cell mass of blastocysts [118]. HAESCs have been shown the potential to differentiate into all three germ layers, such as hepatocytes, cardiomyocytes and neurons [119]. HAESCs have a low mutation rate [120], low immunogenicity [121], and good genetic stability. In addition, these cells also present low tumorigenicity after transplanted into humans [122]. Li et al. transplanted hAESCs derived-RPE cells into the subretinal space of RCS rats, and the degree of retinal cells damage was reduced [123].

From what has been discussed above, hAESCs have the characteristics of abundant sources, genetic stability, low immunogenicity and low tumorigenicity. These cells are attracting a lot of clinical attention in cell transplantation therapy.

The advantages and disadvantages of the above six stem cells are summarized in Table 2.

**Table 2:** Advantages and disadvantages of different stem cells

Stem cell type	Common sources	Advantages	Disadvantages
OECs	Olfactory bulb and olfactory mucosa	Self-renewal Induced differentiation into other cell types	Difficult to obtain Have possibility of carrying donor disease genes
ESCs	The mass of cells in the inner layer of a blastocyst embryo	Self-renewal Potential to differentiate into any type of cells	Have difficulties in inducing differentiation and culture Have risk of inducing tumorigenesis Allogeneic donors face immune rejection and possible hidden genetic defects
MSCs	Bone marrow, adipose tissue, placenta, umbilical cord blood, dental pulp, heart muscle, and liver	Wide sources; Self-renewal Induced differentiation into other types of cells	Have possibility of carrying donor disease genes Allogeneic donors face immune rejection
iPSCs	Reprogram somatic cells into a pluripotent state	Extracted directly from autogenous tissue Fewer ethical issues and lower rejection	Retained epigenetic characteristics of donor cells Have possibility of carrying donor disease genes
RPCs	Inner layer of retina of fetuses	Low immunogenicity and tumorigenicity	Limited ability to proliferate and differentiate Lack of sufficient donor cells
hAESCs	Pluripotent inner cell mass of blastocysts	Good genetic stability; low immunogenicity and tumorigenicity	Have possibility of carrying donor disease genes

### Research progress of stem cells combined with exosomes in the treatment of retinal disorders (Table 3, Fig. 2)

**Table 3:** Treatment of retinal disorders with exosomes from stem cells in recent 5 years

Origin of exosome	Experimental subject	Delivery way	Function	References
Human adipose MSCs, bone marrow MSCs and dental pulp stem cells	Glaucoma rats	Intravitreal injection	Inhibit RGC loss and retinal nerve fibre layer thinning while preserving RGC function	Mead et al. [138]
Human umbilical cord MSCs and allogeneic mice adipose MSCs derived exosomes	Laser induced retinal injury mouse	Intravitreal injection	Down-regulated MCP-1 expression, reduce photoreceptor cell death and retinal injury	Yu et al. [139]
Human bone marrow MSCs derived exosomes	Optic nerve compression rats	Intravitreal injection	Promote RGC survival and axon regeneration, partially prevent RGC axon loss and RGC dysfunction	Mead et al. [133]
Human bone marrow MSCs derived exosomes	Oxygen induced retinopathy mouse	Intravitreal injection	Reduce the severity of retinal ischemia	Moisseiev et al. [140]
Human bone marrow MSCs derived exosomes	Hereditary glaucoma DBA/2J mouse	Intravitreal injection	Protect the optic nerve, preserve the number of RGC and prevent axonal degeneration	Mead et al. [141]
Allogeneic rabbit adipose MSCs derived exosomes	STZ induced diabetic rabbits	Intravenous, intraocular or subconjunctival injection	Increase micRNA-222, anti-inflammatory and antiangiogenic effects	Safwat et al. [129]
Human umbilical cord MSCs derived exosomes	Diabetic rats	Intravitreal injection	Reduce inflammation in the retina	Zhang et al. [130]
Human MSCs derived exosomes	Retinal ischemia rats	Intravitreal injection	Enhance the recovery of retinal function and reduce neuroinflammation and apoptosis	Mathew et al. [142]
Allogeneic rats bone marrow MSCs derived exosomes	Retinal detachment rats	Subretinal injection	Inhibit the apoptosis of photoreceptor cells, maintain the normal retinal structure, anti-inflammatory, neuroprotective and anti-apoptotic effects	Ma et al. [131]

**Fig. 2:**
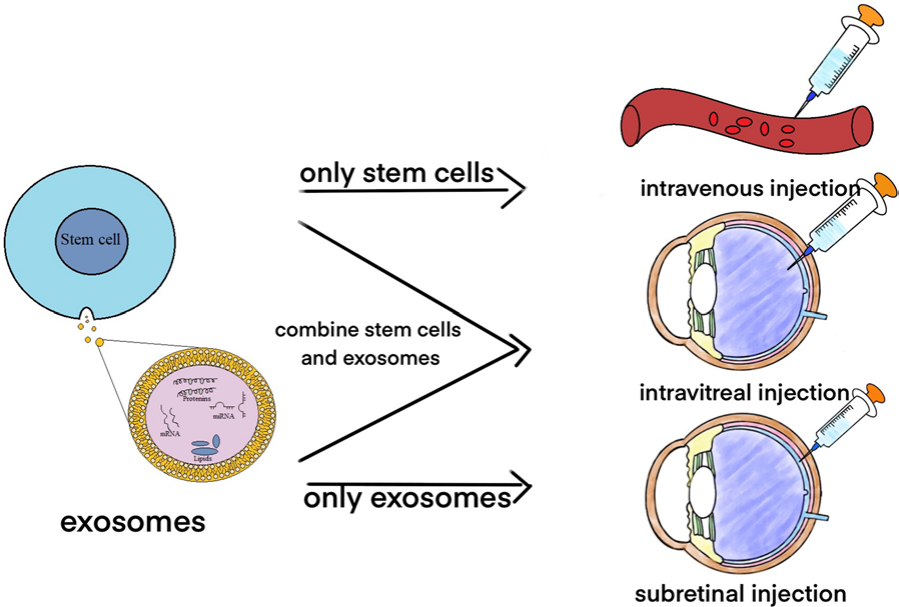
Use stem cells and exosomes to treat retinal disorders. Retinal disorders can be treated with stem cells alone, exosomes alone, or stem cells combined with exosomes through intravenous injection, intravitreal injection, or subretinal injection

Exosomes are extracellular vesicles with a diameter of 30–100 nm and have a lipid bilayer structure. Exosomes can be released by almost all types of cells in the body, such as dendritic cells, lymphocytes, endothelial cells and fibroblasts, and even stem cells and tumor cells. Exosomes can be detected in most body fluids, such as peripheral blood, urine, saliva, ascites and amniotic fluid [124]. Exosomes contain a large number of proteins, lipids, mRNA, miRNA and other signaling molecules [125]. These molecules can reflect the physiological and functional status of secretary cells. For cells with pathological changes, molecular information of these cells can even be obtained by analyzing the exosomes secreted by these cells. It is helpful to the diagnosis and prognosis of some diseases, especially tumors. For example, as a non-coding RNA, miRNA may be abnormally expressed in some tumors and can be used as a biomarker for tumor diagnosis and prognosis [126]. Clinical studies have proved that exosomes have certain effects on ovarian cancer, prostate cancer, bladder cancer, pancreatic cancer and lung cancer. The number of miRNAs in exosomes may be related to the change of tumor size, and the number of serum exosomes in tumor patients may also be related to the stage and grade of tumors. In addition to playing a role in the diagnosis of some cancers, exosomes can also help diagnose non-neoplastic diseases such as kidney disease, chronic hepatitis C, and neurological diseases such as Alzheimer's disease and Parkinson's disease. Exosomes can release mRNA to target cells, allowing the receptor cells to translate exosome related mRNA. Therefore, drugs can be directly transferred to exosomes, or target genes can be encoded into cells that can secrete exosomes, and these exosomes can become ideal drug carrier.

Compared with the therapeutic effect of using stem cells alone, the use of exosomes secreted by stem cells can avoid long-term abnormal cell differentiation and tumorigenesis. Because exosomes are non-cellular components, the possible intravascular embolism after cell transplantation can be avoided. Therefore, the use of stem cell-derived exosomes is expected to be a new therapeutic strategy for tissue damage repair.

Several research groups had used exosomes for the treatment of retinal disorders. He et al. reported that MSCs-derived exosomes can improve the function of RPE under blue light irradiation, and can reduce the degree of retinal damage in animal models of laser retinal injury by down-regulating VEGF-A [127]. A study showed that exosomes derived from adipose MSCs were used as anti-inflammatory mediators to switch macrophages into an alternative activation profile [128]. Safwat et al.'s study on the diabetic rabbit model found that the exosomes derived from MSCs showed anti-inflammatory and anti-angiogenesis effects by increasing micRNA-222 [129]. Similarly, Zhang et al. found when studying diabetic animal models that the expression of miRNA-126 in MSCs-derived exosomes can reduce retinal inflammation by down-regulating the high mobility group BOX1 signaling pathway [130]. In the animal model experiment of retinal detachment established by Ma et al., the exosomes secreted by MSCs can inhibit the apoptosis of photoreceptor cells, maintain the normal retinal structure, and have anti-inflammatory, neuroprotective and anti-apoptotic effects [131]. Pan et al. believed that MSCs-derived exosomes played a neuroprotective role and promoted the survival of RGCs [132]. Furthermore, Mead et al. found that bone marrow MSCs-derived exosomes promoted RGCs survival and axon regeneration, and partially prevented RGCs axon loss and RGCs dysfunction [133]. Ke et al. found that after injection of human ESCs into the vitreous cavity of RCS rats, exosomes derived from ESCs could mediate Muller cells to reverse differentiation into RCS through HSP90 [134]. A study in 2020 showed that exosomes derived from ESCs could preserve RGCs in optic nerve injury mice and rescue the pathological process of tau protein [135]. On the contrary, an interesting study on ESCs-derived exosomes revealed that only microvesicles derived from ESCs could induce Muller cells to differentiate into neuronal cells and achieve regeneration of damaged retina, while ESCs-derived exosomes did not have this effect [136]. Bian et al. injected NPCs-derived exosomes into the subretinal space of RCS rats. The exosomes significantly inhibited microglia activation, reduced photoreceptors apoptosis, and preserved visual function in rats [137].

According to the above experimental results, stem cell-derived exosomes have better effects in regulating immunity, inhibiting inflammation and protecting neuroprotection than stem cell therapy alone in treating retinal disorders.

## Conclusions

Stem cells can preserve the normal immune environment of the retina and promote anti-inflammatory effects. Because stem cells have the ability to migrate and differentiate, they can replace damaged retinal cells and improve the visual function of the experimental group. However, due to the existence of heterogeneous or allogeneic stem cells, immune rejection and ethical issues inevitably appear in many studies. Exosomes alone have been shown to be effective in treating retinal disorders, and some researchers have also combined exosomes with stem cells to discover greater results. Immune rejection is a key issue of stem cells transplantation. The use of autologous stem cells to treat retinal disorders is worth studying. Since human OECs can be safely obtained from olfactory mucosa, researchers may consider using autologous OECs to treat retinal disorders. In addition, exosomes derived from stem cells have been proved to have significant therapeutic effects due to their non-cellular components and secretion, and they will surely become a promising therapeutic method for clinical treatment of retinal disorders. Therefore, improving the quality and quantity of exosomes and achieving industrialized clinical treatment effect is an undoubtedly issues that researchers need to explore in the future. It is noteworthy that the majority of stem cells derived exosomes used in ocular diseases are derived from MSCs in recent years. Based on the characteristics of different stem cells, the study of other stem cells-derived exosomes in retinal disorders, such as OECs-derived exosomes, deserves further attention. Furthermore, exosomes contain a variety of miRNAs, which play a significant role in the diagnosis and prognosis of diseases. Exosomal miRNAs can be used for the prospective diagnosis of early retinal disorders.

## Data Availability

Not applicable.

## Abbreviations

WHO: World Health Organization
AMD: Age-related macular degeneration
DR: Diabetic retinopathy
RP: Retinitis pigmentosa
RPE: Retinal pigment epithelial
CNV: Choroidal neovascularization
anti-VEGF: Anti-vascular endothelial growth factor
AAV: Adeno-associated virus
ONL: Outer nuclear layer
ERG: Electroretinogram
SAM: Senescence accelerated mouse
NPDR: Nonproliferative diabetic retinopathy
PDR: Proliferative diabetic retinopathy
DME: Diabetic macular edema
STZ: Streptozotocin
NOD: Non obese diabetes
RGCs: Retinal ganglion cells
RHO: Rhodopsin
RPGR: Retinitis pigmentosa GTPase regulator
OECs: Olfactory ensheathing cells
NGF: Nerve growth factor
GDNF: Glial cell lineage-derived neurotrophic factor
BDNF: Brain-derived neurotrophic factor
NPY: Neuropeptide Y
BFGF: Basic fibroblast growth factor
NSCs: Neural stem cells
ESCs: Embryonic stem cells
MSCs: Mesenchymal stem cells
iPSCs: Induced pluripotent stem cells
RPCs: Retinal progenitor cells
NPCs: Neural progenitor cells
hRPCs: Human retinal progenitor cells
hAESCs: Human amniotic epithelial stem cells

## References

[1] Steinmetz JD, Bourne RRA, Briant PS, Flaxman SR, Taylor HRB, Jonas JB (2021). Causes of blindness and vision impairment in 2020 and trends over 30 years, and prevalence of avoidable blindness in relation to VISION 2020: the Right to Sight: an analysis for the Global Burden of Disease Study. Lancet Glob Health.

[2] Lim LS, Mitchell P, Seddon JM, Holz FG, Wong TY (2012). Age-related macular degeneration. Lancet.

[3] Buckley K (2011). Age-related macular degeneration. Insight.

[4] Ferrington DA, Kenney MC, Atilano SR, Hurley JB, Brown EE, Ash JD (2021). Mitochondria: the retina's achilles' heel in AMD. Adv Exp Med Biol.

[5] Parmeggiani F, Sorrentino FS, Romano MR, Costagliola C, Semeraro F, Incorvaia C (2013). Mechanism of inflammation in age-related macular degeneration: an up-to-date on genetic landmarks. Mediators Inflamm.

[6] Mitchell P, Liew G, Gopinath B, Wong TY (2018). Age-related macular degeneration. Lancet.

[7] Arrigo A, Bandello F (2021). Molecular features of classic retinal drugs, retinal therapeutic targets and emerging treatments. Pharmaceutics.

[8] Li X, Xu G, Wang Y, Xu X, Liu X, Tang S (2014). Safety and efficacy of conbercept in neovascular age-related macular degeneration: results from a 12-month randomized phase 2 study: AURORA study. Ophthalmology.

[9] Rofagha S, Bhisitkul RB, Boyer DS, Sadda SR, Zhang K, Group S-US (2013). Seven-year outcomes in ranibizumab-treated patients in ANCHOR, MARINA, and HORIZON: a multicenter cohort study (SEVEN-UP). Ophthalmology.

[10] Chakravarthy U, Harding SP, Rogers CA, Downes SM, Lotery AJ, Culliford LA (2013). Alternative treatments to inhibit VEGF in age-related choroidal neovascularisation: 2-year findings of the IVAN randomised controlled trial. Lancet.

[11] Straub LG, Efthymiou V, Grandl G, Balaz M, Challa TD, Truscello L (2019). Antioxidants protect against diabetes by improving glucose homeostasis in mouse models of inducible insulin resistance and obesity. Diabetologia.

[12] Luthy K, Mei D, Fischer B, De Fusco M, Swerts J, Paesmans J (2019). TBC1D24-TLDc-related epilepsy exercise-induced dystonia: rescue by antioxidants in a disease model. Brain.

[13] Li Y, Zhang W, Nguyen VP, Rosen R, Wang X, Xia X (2019). Real-time OCT guidance and multimodal imaging monitoring of subretinal injection induced choroidal neovascularization in rabbit eyes. Exp Eye Res.

[14] Qiu G, Stewart JM, Sadda S, Freda R, Lee S, Guven D (2006). A new model of experimental subretinal neovascularization in the rabbit. Exp Eye Res.

[15] Sherpa RD, Hui SP (2021). An insight on established retinal injury mechanisms and prevalent retinal stem cell activation pathways in vertebrate models. Anim Model Exp Med.

[16] Schmack I, Berglin L, Nie X, Wen J, Kang SJ, Marcus AI (2009). Modulation of choroidal neovascularization by subretinal injection of retinal pigment epithelium and polystyrene microbeads. Mol Vis.

[17] Lyzogubov VV, Tytarenko RG, Liu J, Bora NS, Bora PS (2011). Polyethylene glycol (PEG)-induced mouse model of choroidal neovascularization. J Biol Chem.

[18] Jo YJ, Sonoda KH, Oshima Y, Takeda A, Kohno R, Yamada J (2011). Establishment of a new animal model of focal subretinal fibrosis that resembles disciform lesion in advanced age-related macular degeneration. Investig Ophthalmol Vis Sci.

[19] Baba T, Bhutto IA, Merges C, Grebe R, Emmert D, McLeod DS (2010). A rat model for choroidal neovascularization using subretinal lipid hydroperoxide injection. Am J Pathol.

[20] Martinez B, Peplow PV (2021). MicroRNAs in laser-induced choroidal neovascularization in mice and rats: their expression and potential therapeutic targets. Neural Regen Res.

[21] Olvera-Montano O, Baiza-Duran L, Quintana-Hau JD, Quinonez-Alvarado MG, Zeng W, Gong L (2019). Comparing the efficacy of an anti-human VEGF-a neutralizing antibody versus bevacizumab on a laser-induced choroidal neovascularization (CNV) rhesus monkey model. Drug Des Devel Ther.

[22] Ambati J, Anand A, Fernandez S, Sakurai E, Lynn BC, Kuziel WA (2003). An animal model of age-related macular degeneration in senescent Ccl-2- or Ccr-2-deficient mice. Nat Med.

[23] Combadiere C, Feumi C, Raoul W, Keller N, Rodero M, Pezard A (2007). CX3CR1-dependent subretinal microglia cell accumulation is associated with cardinal features of age-related macular degeneration. J Clin Investig.

[24] Fujihara M, Bartels E, Nielsen LB, Handa JT (2009). A human apoB100 transgenic mouse expresses human apoB100 in the RPE and develops features of early AMD. Exp Eye Res.

[25] Sun LF, Ma Y, Ji YY, Wu Z, Wang YH, Mou H (2021). Circular Rims2 deficiency causes retinal degeneration. Adv Biol (Weinh).

[26] Chen XJ, Zhang ZC, Wang XY, Zhao HQ, Li ML, Ma Y (2020). The circular RNome of developmental retina in mice. Mol Ther Nucleic Acids.

[27] Sun LF, Chen XJ, Jin ZB (2020). Emerging roles of non-coding RNAs in retinal diseases: a review. Clin Exp Ophthalmol.

[28] Chen XJ, Zhang CJ, Wang YH, Jin ZB (2021). Retinal degeneration caused by Ago2 disruption. Investig Ophthalmol Vis Sci.

[29] Shibuya S, Watanabe K, Ozawa Y, Shimizu T (2021). Xanthine oxidoreductase-mediated superoxide production is not involved in the age-related pathologies in Sod1-deficient mice. Int J Mol Sci.

[30] Majji AB, Cao J, Chang KY, Hayashi A, Aggarwal S, Grebe RR (2000). Age-related retinal pigment epithelium and Bruch's membrane degeneration in senescence-accelerated mouse. Investig Ophthalmol Vis Sci.

[31] Kempen JH, O'Colmain BJ, Leske MC, Haffner SM, Klein R, Moss SE (2004). The prevalence of diabetic retinopathy among adults in the United States. Arch Ophthalmol.

[32] Bressler SB, Odia I, Glassman AR, Danis RP, Grover S, Hampton GR (2018). Changes in diabetic retinopathy severity when treating diabetic macular edema with ranibizumab: DRCR.net Protocol I 5-year report. Retina.

[33] Kumar S, Zhuo L (2010). Longitudinal in vivo imaging of retinal gliosis in a diabetic mouse model. Exp Eye Res.

[34] Feit-Leichman RA, Kinouchi R, Takeda M, Fan Z, Mohr S, Kern TS (2005). Vascular damage in a mouse model of diabetic retinopathy: relation to neuronal and glial changes. Investig Ophthalmol Vis Sci.

[35] Makino S, Kunimoto K, Muraoka Y, Mizushima Y, Katagiri K, Tochino Y (1980). Breeding of a non-obese, diabetic strain of mice. Jikken Dobutsu.

[36] Kern TS, Miller CM, Tang J, Du Y, Ball SL, Berti-Matera L (2010). Comparison of three strains of diabetic rats with respect to the rate at which retinopathy and tactile allodynia develop. Mol Vis.

[37] Helfenstein T, Fonseca FA, Ihara SS, Bottos JM, Moreira FT, Pott H (2011). Impaired glucose tolerance plus hyperlipidaemia induced by diet promotes retina microaneurysms in New Zealand rabbits. Int J Exp Pathol.

[38] Olivares AM, Althoff K, Chen GF, Wu S, Morrisson MA, DeAngelis MM (2017). Animal models of diabetic retinopathy. Curr Diab Rep.

[39] Lebherz C, Maguire AM, Auricchio A, Tang W, Aleman TS, Wei Z (2005). Nonhuman primate models for diabetic ocular neovascularization using AAV2-mediated overexpression of vascular endothelial growth factor. Diabetes.

[40] Du SW, Palczewski K (2021). MicroRNA regulation of critical retinal pigment epithelial functions. Trends Neurosci.

[41] Jiang Q, Liu C, Li CP, Xu SS, Yao MD, Ge HM (2020). Circular RNA-ZNF532 regulates diabetes-induced retinal pericyte degeneration and vascular dysfunction. J Clin Investig.

[42] Daiger SP, Sullivan LS, Bowne SJ (2013). Genes and mutations causing retinitis pigmentosa. Clin Genet.

[43] Adackapara CA, Sunness JS, Dibernardo CW, Melia BM, Dagnelie G (2008). Prevalence of cystoid macular edema and stability in oct retinal thickness in eyes with retinitis pigmentosa during a 48-week lutein trial. Retina.

[44] Fujiwara K, Ikeda Y, Murakami Y, Funatsu J, Nakatake S, Tachibana T (2017). Risk factors for posterior subcapsular cataract in retinitis pigmentosa. Investig Ophthalmol Vis Sci.

[45] Triolo G, Pierro L, Parodi MB, De Benedetto U, Gagliardi M, Manitto MP (2013). Spectral domain optical coherence tomography findings in patients with retinitis pigmentosa. Ophthalmic Res.

[46] Dinculescu A, Min SH, Deng WT, Li Q, Hauswirth WW (2014). Gene therapy in the rd6 mouse model of retinal degeneration. Adv Exp Med Biol.

[47] Stronks HC, Dagnelie G (2014). The functional performance of the Argus II retinal prosthesis. Expert Rev Med Devices.

[48] Berson EL, Weigel-DiFranco C, Rosner B, Gaudio AR, Sandberg MA (2018). Association of vitamin A supplementation with disease course in children with retinitis pigmentosa. JAMA Ophthalmol.

[49] Kalloniatis M, Nivison-Smith L, Chua J, Acosta ML, Fletcher EL (2016). Using the rd1 mouse to understand functional and anatomical retinal remodelling and treatment implications in retinitis pigmentosa: a review. Exp Eye Res.

[50] He XY, Zhao CJ, Xu H, Chen K, Bian BS, Gong Y (2021). Synaptic repair and vision restoration in advanced degenerating eyes by transplantation of retinal progenitor cells. Stem Cell Rep.

[51] Winkler PA, Occelli LM, Petersen-Jones SM (2020). Large animal models of inherited retinal degenerations: a review. Cells.

[52] Dinculescu A, Link BA, Saperstein DA (2021). Retinal gene therapy for Usher syndrome: current developments, challenges, and perspectives. Int Ophthalmol Clin.

[53] Massengill MT, Lewin AS (2021). Gene therapy for rhodopsin-associated autosomal dominant retinitis pigmentosa. Int Ophthalmol Clin.

[54] Gumerson JD, Alsufyani A, Yu W, Lei J, Sun X, Dong L (2021). Restoration of RPGR expression in vivo using CRISPR/Cas9 gene editing. Gene Ther.

[55] Ong SS, Patel TP, Singh MS (2019). Optical coherence tomography angiography imaging in inherited retinal diseases. J Clin Med.

[56] Yoshizawa K, Sasaki T, Uehara N, Kuro M, Kimura A, Kinoshita Y (2012). N-ethyl-N-nitrosourea induces retinal photoreceptor damage in adult rats. J Toxicol Pathol.

[57] Vila N, Siblini A, Esposito E, Bravo-Filho V, Zoroquiain P, Aldrees S (2017). Blue-light filtering alters angiogenic signaling in human retinal pigmented epithelial cells culture model. BMC Ophthalmol.

[58] Mookherjee S, Hiriyanna S, Kaneshiro K, Li L, Li Y, Li W (2015). Long-term rescue of cone photoreceptor degeneration in retinitis pigmentosa 2 (RP2)-knockout mice by gene replacement therapy. Hum Mol Genet.

[59] Michalakis S, Koch S, Sothilingam V, Garcia Garrido M, Tanimoto N, Schulze E (2014). Gene therapy restores vision and delays degeneration in the CNGB1(-/-) mouse model of retinitis pigmentosa. Adv Exp Med Biol.

[60] Zhong H, Eblimit A, Moayedi Y, Boye SL, Chiodo VA, Chen Y (2015). AAV8(Y733F)-mediated gene therapy in a Spata7 knockout mouse model of Leber congenital amaurosis and retinitis pigmentosa. Gene Ther.

[61] Koster C, van den Hurk KT, Lewallen CF, Talib M, Ten Brink JB, Boon CJF (2021). The Lrat(-/-) Rat: CRISPR/Cas9 construction and phenotyping of a new animal model for retinitis pigmentosa. Int J Mol Sci.

[62] Xiang L, Chen XJ, Wu KC, Zhang CJ, Zhou GH, Lv JN (2017). miR-183/96 plays a pivotal regulatory role in mouse photoreceptor maturation and maintenance. Proc Natl Acad Sci USA.

[63] Zhang CJ, Xiang L, Chen XJ, Wang XY, Wu KC, Zhang BW (2020). Ablation of mature miR-183 leads to retinal dysfunction in mice. Investig Ophthalmol Vis Sci.

[64] Wu KC, Chen XJ, Jin GH, Wang XY, Yang DD, Li YP (2019). Deletion of miR-182 leads to retinal dysfunction in mice. Investig Ophthalmol Vis Sci.

[65] Sakami S, Kolesnikov AV, Kefalov VJ, Palczewski K (2014). P23H opsin knock-in mice reveal a novel step in retinal rod disc morphogenesis. Hum Mol Genet.

[66] Sancho-Pelluz J, Cui X, Lee W, Tsai YT, Wu WH, Justus S (2019). Mechanisms of neurodegeneration in a preclinical autosomal dominant retinitis pigmentosa knock-in model with a Rho(D190N) mutation. Cell Mol Life Sci.

[67] Reshamwala R, Shah M, Belt L, Ekberg JAK, St John JA (2020). Reliable cell purification and determination of cell purity: crucial aspects of olfactory ensheathing cell transplantation for spinal cord repair. Neural Regen Res.

[68] Minkelyte K, Collins A, Liadi M, Ibrahim A, Li D, Li Y (2021). High-yield mucosal olfactory ensheathing cells restore loss of function in rat dorsal root injury. Cells.

[69] Chou RH, Lu CY, Wei L, Fan JR, Yu YL, Shyu WC (2014). The potential therapeutic applications of olfactory ensheathing cells in regenerative medicine. Cell Transplant.

[70] Murrell W, Feron F, Wetzig A, Cameron N, Splatt K, Bellette B (2005). Multipotent stem cells from adult olfactory mucosa. Dev Dyn.

[71] Au E, Roskams AJ (2003). Olfactory ensheathing cells of the lamina propria in vivo and in vitro. Glia.

[72] Ziegler MD, Hsu D, Takeoka A, Zhong H, Ramon-Cueto A, Phelps PE (2011). Further evidence of olfactory ensheathing glia facilitating axonal regeneration after a complete spinal cord transection. Exp Neurol.

[73] Radtke C, Aizer AA, Agulian SK, Lankford KL, Vogt PM, Kocsis JD (2009). Transplantation of olfactory ensheathing cells enhances peripheral nerve regeneration after microsurgical nerve repair. Brain Res.

[74] Shi X, Kang Y, Hu Q, Chen C, Yang L, Wang K (2010). A long-term observation of olfactory ensheathing cells transplantation to repair white matter and functional recovery in a focal ischemia model in rat. Brain Res.

[75] Ramon-Cueto A, Perez J, Nieto-Sampedro M (1993). In vitro enfolding of olfactory neurites by p75 NGF receptor positive ensheathing cells from adult rat olfactory bulb. Eur J Neurosci.

[76] Boruch AV, Conners JJ, Pipitone M, Deadwyler G, Storer PD, Devries GH (2001). Neurotrophic and migratory properties of an olfactory ensheathing cell line. Glia.

[77] Woodhall E, West AK, Chuah MI (2001). Cultured olfactory ensheathing cells express nerve growth factor, brain-derived neurotrophic factor, glia cell line-derived neurotrophic factor and their receptors. Brain Res Mol Brain Res.

[78] Ubink R, Hokfelt T (2000). Expression of neuropeptide Y in olfactory ensheathing cells during prenatal development. J Comp Neurol.

[79] Cummings DM, Brunjes PC (1995). Migrating luteinizing hormone-releasing hormone (LHRH) neurons and processes are associated with a substrate that expresses S100. Brain Res Dev Brain Res.

[80] Teare KA, Pearson RG, Shakesheff KM, Raisman G, Haycock JW (2003). Alpha-MSH inhibits inflammatory signalling in olfactory ensheathing cells. NeuroReport.

[81] Panni P, Ferguson IA, Beacham I, Mackay-Sim A, Ekberg JA, St John JA (2013). Phagocytosis of bacteria by olfactory ensheathing cells and Schwann cells. Neurosci Lett.

[82] Su Z, Chen J, Qiu Y, Yuan Y, Zhu F, Zhu Y (2013). Olfactory ensheathing cells: the primary innate immunocytes in the olfactory pathway to engulf apoptotic olfactory nerve debris. Glia.

[83] Yin DP, Chen QY, Liu L (2016). Synergetic effects of ciliary neurotrophic factor and olfactory ensheathing cells on optic nerve reparation (complete translation). Neural Regen Res.

[84] Wu MM, Fan DG, Tadmori I, Yang H, Furman M, Jiao XY (2010). Death of axotomized retinal ganglion cells delayed after intraoptic nerve transplantation of olfactory ensheathing cells in adult rats. Cell Transplant.

[85] Huo SJ, Li Y, Raisman G, Yin ZQ (2011). Transplanted olfactory ensheathing cells reduce the gliotic injury response of Muller cells in a rat model of retinitis pigmentosa. Brain Res.

[86] Huo SJ, Li YC, Xie J, Li Y, Raisman G, Zeng YX (2012). Transplanted olfactory ensheathing cells reduce retinal degeneration in Royal College of Surgeons rats. Curr Eye Res.

[87] Xie J, Huo S, Li Y, Dai J, Xu H, Yin ZQ (2017). Olfactory ensheathing cells inhibit gliosis in retinal degeneration by downregulation of the muller cell notch signaling pathway. Cell Transplant.

[88] Xue L, Zeng Y, Li Q, Li Y, Li Z, Xu H (2017). Transplanted olfactory ensheathing cells restore retinal function in a rat model of light-induced retinal damage by inhibiting oxidative stress. Oncotarget.

[89] Xie J, Li Y, Dai J, He Y, Sun D, Dai C (2019). Olfactory ensheathing cells grafted into the retina of RCS rats suppress inflammation by down-regulating the JAK/STAT pathway. Front Cell Neurosci.

[90] Hua Wang Y, Wu Wang D, Qin YZ (2020). Synergistic protection of RGCs by olfactory ensheathing cells and alpha-crystallin through regulation of the Akt/BAD Pathway. J Fr Ophtalmol.

[91] Zhai W, Gao L, Qu L, Li Y, Zeng Y, Li Q (2020). Combined transplantation of olfactory ensheathing cells with rat neural stem cells enhanced the therapeutic effect in the retina of RCS rats. Front Cell Neurosci.

[92] Leach LL, Clegg DO (2015). Concise review: making stem cells retinal: methods for deriving retinal pigment epithelium and implications for patients with ocular disease. Stem Cells.

[93] da Cruz L, Fynes K, Georgiadis O, Kerby J, Luo YH, Ahmado A (2018). Phase 1 clinical study of an embryonic stem cell-derived retinal pigment epithelium patch in age-related macular degeneration. Nat Biotechnol.

[94] Chao JR, Lamba DA, Klesert TR, Torre A, Hoshino A, Taylor RJ (2017). Transplantation of human embryonic stem cell-derived retinal cells into the subretinal space of a non-human primate. Transl Vis Sci Technol.

[95] Liu Z, Ilmarinen T, Tan GSW, Hongisto H, Wong EYM, Tsai ASH (2021). Submacular integration of hESC-RPE monolayer xenografts in a surgical non-human primate model. Stem Cell Res Ther.

[96] Scalinci SZ, Scorolli L, Corradetti G, Domanico D, Vingolo EM, Meduri A (2011). Potential role of intravitreal human placental stem cell implants in inhibiting progression of diabetic retinopathy in type 2 diabetes: neuroprotective growth factors in the vitreous. Clin Ophthalmol.

[97] Tomita M, Adachi Y, Yamada H, Takahashi K, Kiuchi K, Oyaizu H (2002). Bone marrow-derived stem cells can differentiate into retinal cells in injured rat retina. Stem Cells.

[98] Gong L, Wu Q, Song B, Lu B, Zhang Y (2008). Differentiation of rat mesenchymal stem cells transplanted into the subretinal space of sodium iodate-injected rats. Clin Exp Ophthalmol.

[99] Shaw LC, Neu MB, Grant MB (2011). Cell-based therapies for diabetic retinopathy. Curr Diab Rep.

[100] Abdi R, Fiorina P, Adra CN, Atkinson M, Sayegh MH (2008). Immunomodulation by mesenchymal stem cells: a potential therapeutic strategy for type 1 diabetes. Diabetes.

[101] Nauta AJ, Fibbe WE (2007). Immunomodulatory properties of mesenchymal stromal cells. Blood.

[102] Chen S, Cui G, Peng C, Lavin MF, Sun X, Zhang E (2018). Transplantation of adipose-derived mesenchymal stem cells attenuates pulmonary fibrosis of silicosis via anti-inflammatory and anti-apoptosis effects in rats. Stem Cell Res Ther.

[103] Ortiz-Virumbrales M, Menta R, Perez LM, Lucchesi O, Mancheno-Corvo P, Avivar-Valderas A (2020). Human adipose mesenchymal stem cells modulate myeloid cells toward an anti-inflammatory and reparative phenotype: role of IL-6 and PGE2. Stem Cell Res Ther.

[104] Peng BY, Dubey NK, Mishra VK, Tsai FC, Dubey R, Deng WP (2018). Addressing stem cell therapeutic approaches in pathobiology of diabetes and its complications. J Diabetes Res.

[105] Song MJ, Bharti K (2016). Looking into the future: using induced pluripotent stem cells to build two and three dimensional ocular tissue for cell therapy and disease modeling. Brain Res.

[106] Mellough CB, Sernagor E, Moreno-Gimeno I, Steel DH, Lako M (2012). Efficient stage-specific differentiation of human pluripotent stem cells toward retinal photoreceptor cells. Stem Cells.

[107] Stadtfeld M, Nagaya M, Utikal J, Weir G, Hochedlinger K (2008). Induced pluripotent stem cells generated without viral integration. Science.

[108] Buchholz DE, Pennington BO, Croze RH, Hinman CR, Coffey PJ, Clegg DO (2013). Rapid and efficient directed differentiation of human pluripotent stem cells into retinal pigmented epithelium. Stem Cells Transl Med.

[109] Kim D, Kim CH, Moon JI, Chung YG, Chang MY, Han BS (2009). Generation of human induced pluripotent stem cells by direct delivery of reprogramming proteins. Cell Stem Cell.

[110] Gonzalez-Cordero A, Goh D, Kruczek K, Naeem A, Fernando M, Kleine Holthaus SM (2018). Assessment of AAV vector tropisms for mouse and human pluripotent stem cell-derived RPE and photoreceptor cells. Hum Gene Ther.

[111] Michelet F, Balasankar A, Teo N, Stanton LW, Singhal S (2020). Rapid generation of purified human RPE from pluripotent stem cells using 2D cultures and lipoprotein uptake-based sorting. Stem Cell Res Ther.

[112] Lingam S, Liu Z, Yang B, Wong W, Parikh BH, Ong JY (2021). cGMP-grade human iPSC-derived retinal photoreceptor precursor cells rescue cone photoreceptor damage in non-human primates. Stem Cell Res Ther.

[113] Wang Y, Tang Z, Gu P (2020). Stem/progenitor cell-based transplantation for retinal degeneration: a review of clinical trials. Cell Death Dis.

[114] Baranov PY, Tucker BA, Young MJ (2014). Low-oxygen culture conditions extend the multipotent properties of human retinal progenitor cells. Tissue Eng Part A.

[115] Wang Z, Gao F, Zhang M, Zheng Y, Zhang F, Xu L (2020). Intravitreal injection of human retinal progenitor cells for treatment of retinal degeneration. Med Sci Monit.

[116] Liu Y, Chen SJ, Li SY, Qu LH, Meng XH, Wang Y (2017). Long-term safety of human retinal progenitor cell transplantation in retinitis pigmentosa patients. Stem Cell Res Ther.

[117] Ballios BG, Cooke MJ, Donaldson L, Coles BL, Morshead CM, van der Kooy D (2015). A hyaluronan-based injectable hydrogel improves the survival and integration of stem cell progeny following transplantation. Stem Cell Rep.

[118] Yang PJ, Yuan WX, Liu J, Li JY, Tan B, Qiu C (2018). Biological characterization of human amniotic epithelial cells in a serum-free system and their safety evaluation. Acta Pharmacol Sin.

[119] Li B, Zhang Q, Sun J, Lai D (2019). Human amniotic epithelial cells improve fertility in an intrauterine adhesion mouse model. Stem Cell Res Ther.

[120] Miki T (2018). Stem cell characteristics and the therapeutic potential of amniotic epithelial cells. Am J Reprod Immunol.

[121] Strom SC, Gramignoli R (2016). Human amnion epithelial cells expressing HLA-G as novel cell-based treatment for liver disease. Hum Immunol.

[122] Qiu C, Ge Z, Cui W, Yu L, Li J (2020). Human amniotic epithelial stem cells: a promising seed cell for clinical applications. Int J Mol Sci.

[123] Li J, Qiu C, Wei Y, Yuan W, Liu J, Cui W (2021). Human amniotic epithelial stem cell-derived retinal pigment epithelium cells repair retinal degeneration. Front Cell Dev Biol.

[124] Simons M, Raposo G (2009). Exosomes–vesicular carriers for intercellular communication. Curr Opin Cell Biol.

[125] Hunter MP, Ismail N, Zhang X, Aguda BD, Lee EJ, Yu L (2008). Detection of microRNA expression in human peripheral blood microvesicles. PLoS ONE.

[126] Lu J, Getz G, Miska EA, Alvarez-Saavedra E, Lamb J, Peck D (2005). MicroRNA expression profiles classify human cancers. Nature.

[127] He GH, Zhang W, Ma YX, Yang J, Chen L, Song J (2018). Mesenchymal stem cells-derived exosomes ameliorate blue light stimulation in retinal pigment epithelium cells and retinal laser injury by VEGF-dependent mechanism. Int J Ophthalmol.

[128] Lo Sicco C, Reverberi D, Balbi C, Ulivi V, Principi E, Pascucci L (2017). Mesenchymal stem cell-derived extracellular vesicles as mediators of anti-inflammatory effects: endorsement of macrophage polarization. Stem Cells Transl Med.

[129] Safwat A, Sabry D, Ragiae A, Amer E, Mahmoud RH, Shamardan RM (2018). Adipose mesenchymal stem cells-derived exosomes attenuate retina degeneration of streptozotocin-induced diabetes in rabbits. J Circ Biomark.

[130] Zhang W, Wang Y, Kong Y (2019). Exosomes derived from mesenchymal stem cells modulate miR-126 to ameliorate hyperglycemia-induced retinal inflammation via targeting HMGB1. Invest Ophthalmol Vis Sci.

[131] Ma M, Li B, Zhang M, Zhou L, Yang F, Ma F (2020). Therapeutic effects of mesenchymal stem cell-derived exosomes on retinal detachment. Exp Eye Res.

[132] Pan D, Chang X, Xu M, Zhang M, Zhang S, Wang Y (2019). UMSC-derived exosomes promote retinal ganglion cells survival in a rat model of optic nerve crush. J Chem Neuroanat.

[133] Mead B, Tomarev S (2017). Bone marrow-derived mesenchymal stem cells-derived exosomes promote survival of retinal ganglion cells through miRNA-dependent mechanisms. Stem Cells Transl Med.

[134] Ke Y, Fan X, Hao R, Dong L, Xue M, Tan L (2021). Human embryonic stem cell-derived extracellular vesicles alleviate retinal degeneration by upregulating Oct4 to promote retinal Muller cell retrodifferentiation via HSP90. Stem Cell Res Ther.

[135] Seyedrazizadeh SZ, Poosti S, Nazari A, Alikhani M, Shekari F, Pakdel F (2020). Extracellular vesicles derived from human ES-MSCs protect retinal ganglion cells and preserve retinal function in a rodent model of optic nerve injury. Stem Cell Res Ther.

[136] Peng Y, Baulier E, Ke Y, Young A, Ahmedli NB, Schwartz SD (2018). Human embryonic stem cells extracellular vesicles and their effects on immortalized human retinal Muller cells. PLoS ONE.

[137] Bian B, Zhao C, He X, Gong Y, Ren C, Ge L (2020). Exosomes derived from neural progenitor cells preserve photoreceptors during retinal degeneration by inactivating microglia. J Extracell Vesicles.

[138] Mead B, Hill LJ, Blanch RJ, Ward K, Logan A, Berry M (2016). Mesenchymal stromal cell-mediated neuroprotection and functional preservation of retinal ganglion cells in a rodent model of glaucoma. Cytotherapy.

[139] Yu B, Shao H, Su C, Jiang Y, Chen X, Bai L (2016). Exosomes derived from MSCs ameliorate retinal laser injury partially by inhibition of MCP-1. Sci Rep.

[140] Moisseiev E, Anderson JD, Oltjen S, Goswami M, Zawadzki RJ, Nolta JA (2017). Protective effect of intravitreal administration of exosomes derived from mesenchymal stem cells on retinal ischemia. Curr Eye Res.

[141] Mead B, Ahmed Z, Tomarev S (2018). Mesenchymal stem cell-derived small extracellular vesicles promote neuroprotection in a genetic DBA/2J mouse model of glaucoma. Investig Ophthalmol Vis Sci.

[142] Mathew B, Ravindran S, Liu X, Torres L, Chennakesavalu M, Huang CC (2019). Mesenchymal stem cell-derived extracellular vesicles and retinal ischemia-reperfusion. Biomaterials.

